# Accounting for clustering for self-reported outcomes in the design and analysis of population-based surveys: A case study of estimation of prevalence of epilepsy in Nairobi, Kenya

**DOI:** 10.3389/frma.2025.1583476

**Published:** 2025-09-01

**Authors:** Daniel M. Mwanga, Isaac C. Kipchirchir, George O. Muhua, Charles R. Newton, Damazo T. Kadengye

**Affiliations:** ^1^Department of Mathematics, University of Nairobi, Nairobi, Kenya; ^2^Data Synergy and Evaluations, African Population and Health Research Center, Nairobi, Kenya; ^3^Department of Psychiatry, University of Oxford, Oxford, United Kingdom; ^4^Neurosciences Unit, Kenya Medical Research Institute, Wellcome Trust Research Programme, Kilifi, Kenya

**Keywords:** prevalence, epilepsy, interviewer effects, clustering, hierarchical structure, multi-level modeling, population-based surveys

## Abstract

Population-based surveys are common for estimation of important public health metrics such as prevalence. Often, survey data tend to have a hierarchical structure where households are clustered within villages or sites and interviewers are assigned specific locations to conduct the survey. Self-reported outcomes such as diagnosis of diseases like epilepsy present more complex structure, where interviewer or physician-related effects may bias the results. Standard estimation techniques that ignore clustering may lead to underestimated standard errors and overconfident inferences. In this paper, we examine these effects for estimation of prevalence of epilepsy in a two-stage population-based survey in Nairobi and we discuss how clustering can be taken into account in design and analysis of population-based prevalence studies. We used data from the Epilepsy Pathway Innovation in Africa project conducted in Nairobi and simulated attrition levels at 10% and 20% assuming missing at random (MAR) mechanism. Attrition was accounted for using sequential k-nearest neighbor method. We adjusted the expected prevalence based on clustering at multiple levels, such as site, interviewer and household using a random effects model. Intraclass correlation (ICC) > 0.1 indicated presence of substantial clustering. We report point estimates with 95% confidence interval (CI). Crude prevalence of epilepsy was 9.40 cases per 1,000 people (95% CI: 8.60–10.20). There was substantial clustering at household level (ICC = 0.397), interviewer level (ICC = 0.101) and site level (ICC = 0.070). Prevalence adjusted for clustering at household, interviewer and site was 9.15/1,000 (95% CI 7.11–11.20). Overall, not accounting for clustering was associated with underestimation of standard errors. Not accounting for attrition on the other hand led to underestimation of prevalence. Imputation of the missing data due to attrition mitigated the attrition bias under appropriate assumptions. Accounting for clustering, particularly household, interviewer and site levels, is critical for valid estimation of standard errors in population-based surveys. Rigorous training and pre-survey testing can minimize measurement error in self-reported outcomes. Attrition can lead to underestimation of prevalence if not properly addressed. Attrition bias can be minimized by conducting targeted mobilization of participants to improve response rates and using statistical methods such as multiple imputation or machine learning-based imputation methods to address it.

## 1 Introduction

Prevalence is an important metric for public health surveillance. Accurate estimation of disease prevalence is crucial for effective public policy decisions such as allocation of resources. For diseases such as epilepsy, prevalence is often estimated using two stages. In the first stage, residents of a target population area are screened using a standardized questionnaire by a trained team of field interviewers who interview a head of the household or the available adult member of the household in the absence of the head of the household. In the second stage, those that are screened as possible cases in the first stage are invited to a nearby healthcare facility for confirmation of diagnosis by a trained physician, such as a neurologist. Both stages rely on self reports, which are prone to biases due to measurement errors, recall biases, and contextual factors leading to variability of estimates at several levels. Two of the most common sources of variability that can affect self-reported data are interviewer effects and geographical site level clustering (West and Li, 2019).

Interviewer effects arise when differences in interviewer characteristics (such as, demeanor, skill, or demographic traits) influence how respondents answer questions. While interviewer variability may be inevitable in practice, biases related to differences in interviewer characteristics needs to be minimized. Site clustering effects occur when respondents within the same geographic or organizational site share similar conditions that may influence their responses. These may include factors such as access to healthcare, cultural norms, exposure to similar levels of awareness or environmental risk factors that influence their behavior in certain situations. Ignoring these effects can lead to biased prevalence estimates and underestimated uncertainty, thereby undermining the utility of the findings.

Previous studies have shown that interviewers may contribute to measurement differences through variations in how they individually administer survey questions or influence responses in one way or another (Olson et al., 2020; Lipps and Lutz, 2017), which may impact the estimation of parameters (Harling et al., 2019). Further, epilepsy is diagnosed by a physician taking history from a patient, and relying on the self-reported responses to make a determination of diagnosis and the type of epilepsy. In this study, we refer to the potential differences arising from how interviewers ask the questions, as interviewer level effects. For instance, if there exist differences among interviewers, participants screened by one interviewer could be more likely to respond to screening questions in a particular way or have a similar decision about participation in the study, thereby influencing response rate (McGovern et al., 2015; Durrant et al., 2010). Other levels of clustering could include at community level or at household level. Households from one geographical site are more likely to have similar characteristics related to the condition under study because they could be exposed to similar conditions.

Different levels of clustering could be modeled using multiple approaches. Studies show that mixed-effects models provide flexibility of modeling hierarchical clusters (Snijders and Bosker, 2011) including interviewer related random effects (McGovern et al., 2015). In this paper, we discuss the importance of accounting for these effects during design and analysis of prevalence surveys. The focus of this study, is to analyze the effect of different levels of clustering on the estimation of the prevalence of epilepsy. By utilizing mixed-effects models, we aim to examine the effect of interviewer-level clustering and examine its impact on prevalence estimates. We also examine hierarchical modeling of different levels of clustering. The findings will provide insights into the importance of accounting for clustering effects in survey-based studies to improve measurement of metrics such as prevalence, especially for conditions where diagnosis relies heavily on self-reported data. The implications of this research will contribute to improving the accuracy and reliability of prevalence estimates, informing better study designs, and enhancing data collection protocols and metrics in epidemiological research.

## 2 Materials and methods

### 2.1 Hierarchical model structure

Consider a hierarchical data structure where individuals are clustered within households and the interviewer assigned to screen that household and interviewers are also clustered within sites. We define the hierarchical structure of the data as follows:

- *Y*_*ijk*_: The binary outcome for the *k*-th individual screened by the *j*-th interviewer within the *i*-th site.
- **X**_*ijk*_: Covariates associated with the *k*-th individual screened by the *j*-th interviewer within the *i*-th site.
- The binary outcome *Y*_*ijk*_ follows a Bernoulli distribution: *Y*_*ijk*_~Bernoulli(π_*ijk*_), where π_*ijk*_ is the probability of individual *k* screened by interviewer *j* in site *i* being diagnosed with epilepsy. Thus, the event of interest in this analysis is epilepsy diagnosis.

The probability mass function of the Bernoulli distribution is given by


(1) $$\begin{array}{l} P\left(Y_{ijk}=y_{ijk}\right)=\pi_{ijk}^{y}{\left(1-\pi_{ijk}\right)}^{1-y},\;\text{for }y_{ijk}\in \left\{0,1\right\},0<\pi <1 \end{array}$$

Where

- *y* = 1 represents the event occurring, and *y* = 0 represents event not occuring.
- π_*ijk*_ is the probability of the event (i.e., *P*(*Y*_*ijk*_ = 1) = π_*ijk*_).
- 1−π_*ijk*_ is the probability of the event not occurring (i.e., *P*(*Y*_*ijk*_ = 0) = 1−π_*ijk*_).

In the context of our model, π_*ijk*_ is modeled as a logistic function of individual-level covariates **X**_*ijk*_ and random effects for the site and interviewer levels. This is represented as


(2) $$\begin{array}{l} \pi_{ijk}={\text{logit}}^{-1}\left(\beta_{0}+{\text{X}}_{ijk}\beta +u_{i}+v_{ij}\right) \end{array}$$

Where

- β_0_ is the intercept.
- **X**_*ijk*_ is the covariates for individual *i* screened by interviewer *j* within site *i*.
- ***β*** is a vector of coefficients for individual-level covariates.
- *u*_*i*_ is the random effect for the site level ($u_{i}\sim N\left(0,\sigma_{u}^{2}\right)$).
- *v*_*ij*_ is the random effect for the interviewer level ($v_{ij}\sim N\left(0,\sigma_{v}^{2}\right)$).

We assume that the random effect terms *u*_*i*_ and *v*_*ij*_ are independent and that $u_{i}\sim N\left(0,\sigma_{u}^{2}\right)$ and $v_{ij}\sim N\left(0,\sigma_{v}^{2}\right)$, and the residual errors $\epsilon_{ijk}\sim N\left(0,\sigma_{\epsilon }^{2}\right)$, independent of the random effects.

This hierarchical model allows for the estimation of prevalence while accounting for intraclass correlation (ICC) at various clustering levels. The ICC quantifies the proportion of total variance in the outcome that is attributable to clustering at each hierarchical level. In our context, a higher ICC indicates that individuals within the same cluster are more similar with respect to the epilepsy diagnosis outcome than individuals from different cluster. Similarly, ICCs in a hierarchical model structure reflect how much of the variation is due to differences between the various levels.

**Figure 1:**
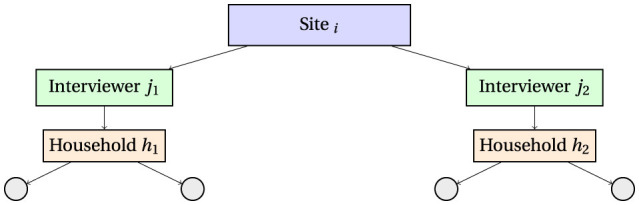
Hierarchical data structure illustrating clustering: individuals are nested within households, households are assigned to interviewers, and interviewers are nested within sites. The intraclass correlation coefficient (ICC) quantifies the proportion of variance attributable to each clustering level.

This is represented in Figure 1.

### 2.2 Model likelihood and estimation

The likelihood function for the binary outcome is the product of the individual Bernoulli likelihoods. For a random effect model with multiple levels, the joint likelihood is given as


(3) $$\begin{array}{l} L\left(\beta_{0},\beta ,\sigma_{u}^{2},\sigma_{v}^{2}\right)=\overset{I}{\underset{i=1}{\prod }}\overset{J_{i}}{\underset{j=1}{\prod }}\overset{K_{ij}}{\underset{k=1}{\prod }}\pi_{ijk}^{y_{ijk}}{\left(1-\pi_{ijk}\right)}^{1-y_{ijk}}\cdot f\left(u_{i},v_{ij};\sigma_{u}^{2},\sigma_{v}^{2}\right) \end{array}$$

where $f\left(u_{i},v_{ij};\sigma_{u}^{2},\sigma_{v}^{2}\right)$ is the joint density of the random effects, assumed to be independent and follow normal distribution


(4) $$\begin{array}{l} f\left(u_{i},v_{ij};\sigma_{u}^{2},\sigma_{v}^{2}\right)=\frac{1}{\left(2\pi \right)\sigma_{u}\sigma_{v}}\exp\left(-\frac{1}{2}\left(\frac{u_{i}^{2}}{\sigma_{u}^{2}}+\frac{v_{ij}^{2}}{\sigma_{v}^{2}}\right)\right) \end{array}$$

The parameters β_0_, ***β***, $\sigma_{u}^{2}$, and $\sigma_{v}^{2}$ are estimated by maximizing this likelihood using restricted maximum likelihood (REML) or full maximum likelihood (ML) methods, implemented in standard statistical software such as R's lme4 package or glmer() function for logistic mixed models.

### 2.3 Expected value and prevalence calculation

Expected value of *Y*_*ijk*_, is the probability of individual *k*, screened by interviewer *j* in site *i*, being screened as having epilepsy.


(5) $$\begin{array}{l} E\left[Y_{ijk}\right]=P\left(Y_{ijk}=1\right)={\text{logit}}^{-1}\left(\beta_{0}+{\text{X}}_{ijk}\beta +u_{i}+v_{ij}\right) \end{array}$$

Thus, the prevalence of epilepsy in the population adjusted for clustering, represented by θ, can be estimated as the average of the individual probabilities. More precisely,


(6) $$\begin{array}{l} \hat{\theta }=\frac{1}{N}\overset{I}{\underset{i=1}{\sum }}\overset{J_{i}}{\underset{j=1}{\sum }}\overset{K_{ij}}{\underset{k=1}{\sum }}{\hat{\pi }}_{ijk} \end{array}$$

Where *N* is the total number of individuals in the survey.

### 2.4 Hierarchical variance decomposition

Since the variance is influenced by the random effects, the total variance of the outcome is the sum of the variances due to each hierarchical level


(7) $$\begin{array}{l} \text{Var}\left(Y_{ijk}\right)=\text{Var}\left(u_{i}\right)+\text{Var}\left(v_{ij}\right)=\sigma_{u}^{2}+\sigma_{v}^{2} \end{array}$$

This hierarchical variance decomposition allows us to appropriately account for the variation in the outcome at both the site and interviewer levels.

### 2.5 Prevalence estimation with random effects

Since the focus of the paper is to estimate prevalence of epilepsy while accounting for the hierarchical clustering structure of the data, consider a model with no covariates given by


(8) $$\begin{array}{l} Y_{ijk}=\beta_{0}+\epsilon_{ijk} \end{array}$$

where

*Y*_*ijk*_ is the outcome for individual *k* screened by interviewer *j* in site *i*, β_0_ the intercept, and ϵ_*ijk*_ the residual error with 𝔼[ϵ_*ijk*_] = 0.

Taking the expectation on both sides,


$$E\left[Y_{ijk}\right]=E\left[\beta_{0}+\epsilon_{ijk}\right]$$

then


$$E\left[Y_{ijk}\right]=\beta_{0}$$

Thus, the intercept β_0_ in the model with only the outcome and no covariates represents the mean of the outcome variable 𝔼[*Y*_*ijk*_]. For binary outcomes, this is equivalent to the probability of the event, which when averaged over the total number of people in the survey, is equivalent to the prevalence.

Now, if we add a random effects terms (e.g., to account for clustering or group-level variability), the model becomes,


(9) $$\begin{array}{l} Y_{ijk}=\beta_{0}+u_{i}+v_{ij}+\epsilon_{ijk} \end{array}$$

where

- $u_{i}\sim N\left(0,\sigma_{u}^{2}\right)$ is the random effect for cluster *i* (site level clustering),
- $v_{ij}\sim N\left(0,\sigma_{v}^{2}\right)$ is the random effect for cluster *j* (interviewer level clustering within sites), and
- $\epsilon_{ijk}\sim N\left(0,\sigma_{\epsilon }^{2}\right)$ is the residual term.

Taking the expectation over both the random effects *u*_*i*_ and *v*_*ij*_ and residual error ϵ_*ijk*_,


$$E\left[Y_{ijk}\right]=E\left[\beta_{0}+u_{i}+v_{ij}+\epsilon_{ijk}\right]$$

Since 𝔼[*u*_*i*_] = 0, 𝔼[*v*_*ij*_] = 0 and 𝔼[ϵ_*ijk*_] = 0:


$$E\left[Y_{ij}\right]=\beta_{0}$$

Here, β_0_ still represents the overall mean (or prevalence for binary outcomes). However, the inclusion of the random effect terms allows for adjustment of the β_0_ due to the clustering at those levels. The adjustment occurs because random effects captures group-level deviations from the overall mean, ensuring that β_0_ is not biased by these deviations.

### 2.6 Empirical study design and dataset description

The study is based on data from the Epilepsy Pathway Innovation in Africa (EPInA) project conducted in Nairobi Urban Health and Demographic Surveillance System (NUHDSS) (EPInA, 2020). The detailed description of the NUHDSS has been published elsewhere (Wamukoya et al., 2020; Emina et al., 2011; Mwanga et al., 2024). In summary, the NUHDSS is comprised of two urban informal settlements namely Viwandani and Korogocho. Like most other urban slums in Nairobi, Viwandani and Korogocho are characterized by lack of basic infrastructure, poor sanitation, overcrowding, high unemployment rate, poverty, and inadequate health infrastructure. While both are urban informal residential areas in Nairobi, there exist some differences between them. Viwandani is located in the industrial area of Nairobi and consists of smaller households with less than three members with majority being households occupied by individuals working in the surrounding companies. Residents in Viwandani are more mobile, with higher migration rates than Korogocho. Korogocho on the other hand consists of a more stable population with households whose residents have stayed there all their life. Households tend to be larger in this site compared to households in Viwandani.

The EPInA prevalence study was designed as a two-stage population-based survey (census). In the first stage, trained interviewers screened all residents in the NUHDSS for possible cases of epilepsy using a standard screening questionnaire (Placencia et al., 1992). Interviewers were assigned to the two sites randomly. In the second stage, those that screened positive in the first stage were invited for confirmation of diagnosis by a neurologist at a nearby local health facility. Clustering levels considered include location (site), interviewer level and at household level. Table 1 describes the different levels of clusters considered in the study.

**Table 1:** Cluster variables considered in the study.

**Variable**	**Description**	**Measurement**
Site	Variable describing residential location in the NUHDSS	0 = Viwandani, 1 = Korogocho. This variable is included for the purpose of modeling hierarchical clustering
Village	Variable indicating villages within the two locations in the HDSS	*Villages in Viwandani*; Paradise A, Paradise B, Paradise C, Sinai Original, Sinai Reli, Jamaica, Lunga Lunga Center, Milimani, Donholm, Riverside, Kingstone, Uchumi; *Villages in Korogocho*; Korogocho B, Korogocho A, Grogon A, Grogon B, Gitathuru, Highridge, Nyayo, Kisumu Ndogo;
Household ID	Unique ID assigned to each household in the NUHDSS	Unique ID assigned to each household in the NUHDSS. More than one individual could belong to 1 household. Household sizes ranged from 1 to 15 members
Interviewer ID	Unique ID assigned to each interviewer	Unique ID assigned to each interviewer. Interviewers were assigned to the sites. This means that, an interviewer assigned to Viwandani site only interviewed participants in Viwandani and those assigned to Korogocho only interviewed those in Korogocho

### 2.7 Statistical analysis

Clusters are considered at multiple levels including household, site and interviewer-related clustering. First, we descriptively examine the prevalence estimates disaggregated by different cluster levels. Differences are tested using χ^2^ at 5% level of significance. Where differences are significant, it means potential clustering exists. We assumed that if clustering is absent, then observations within and between clusters are independent.

We estimated the effect of clustering of epilepsy using the generalized linear mixed models for logistic regression. In this model, we introduced random effects to model the correlation within clusters. The random effects are included to capture the nested hierarchical structure. Generally, in mixed effects modeling, the inclusion of random effects allows for the estimation of both fixed effects (covariate effects) and random effects (cluster-level effects) simultaneously, providing a more comprehensive understanding of the relationship between the covariates and the outcome, while accounting for clustering. Mixed models are flexible and can fit within other modeling approaches such as generalized linear models (West et al., 2022; Luke, 2017).

Since the focus of our study is estimation of prevalence of epilepsy, as outlined previously, we fitted a model with only the outcome and the random effect terms. Prevalence is estimated by the coefficient of the intercept. The coefficient of intercept of the model without the covariates (empty model) is the prevalence estimate when no accounting for clustering is considered. When random effects are added, the new coefficient of the intercept is the new estimate of prevalence when clustering is taken into account. Likelihood ratio tests are used to compare the model with fixed effects and the models with random effects. Other model diagnostic tests included evaluating the models Akaike information criterion (AIC) and Bayesian information creiterion (BIC), where lower values of AIC indicate a better fit. Sensitivity analysis for the various models included assessment of the robustness of key estimates such as prevalence and ICC across the various model structures.

In practice, longitudinal or multi-stage study designs often presents data with missing data due to attrition. We have included this by simulating 10% and 20% attrition levels to examine how it affects prevalence and how estimates change when both clustering and attrition are taken into account. Missing data mechanisms are commonly classified as missing completely at random (MCAR), missing at random (MAR), or missing not at random (MNAR) as defined by Rubin (1976). In this study, we assume a missing at random (MAR) mechanism, where the probability of missingness depends only on observed data and not on the unobserved (missing) values themselves. This assumption underlies commonly used approaches such as multiple imputation (MI) and inverse probability weighting (IPW). To address missingness due to attrition, we employed the sequential k-nearest neighbors (sKNN) algorithm. The sKNN method was chosen due to its practical advantages when estimating random effects models, particularly for calculating intraclass correlation coefficients (ICCs), which can be challenging to obtain reliably from multiply imputed datasets. Additionally, being non-parametric method, sKNN does not require distributional assumptions and has been shown to perform well in such designs (Tutz and Ramzan, 2015; Lai et al., 2019), particularly in scenarios where the MAR assumption may not be plausible or when computational complexity is a concern.

### 2.8 Ethical consideration

The study was approved by Scientific Ethics Review Unit (SERU) at the Kenya Medical Research Institute (KEMRI) (Reference Number: KEMRI/RES/7/3/1). Written informed consent was obtained from all study participants.

## 3 Results and discussion

### 3.1 Empirical results

#### 3.1.1 Descriptive statistics

The two sites, Korogocho and Viwandani, form the NUHDSS (Beguy et al., 2015), and in each site there exist several villages. Table 2 presents prevalence estimates by site and by village. Since the current study did a census (visited all households), the villages listed in Table 2 constitute all the villages in each of the two sites under NUHDSS.

**Table 2:** Prevalence by site and by village.

	**$\hat{\theta }$/1,000**	**σ**	**LCB (L)**	**UCB (U)**
All	9.40	0.41	8.60	10.20
*Site*	(*p* < 0.001)			
Viwandani	8.32	0.49	7.36	9.27
Korogocho	11.15	0.72	9.75	12.56
*Villages in Viwandani*	(*p* < 0.001)			
Paradise A	10.62	2.64	5.44	15.80
Paradise B	23.05	3.56	16.07	30.02
Paradise C	5.32	2.01	1.39	9.26
Sinai Original	7.73	1.77	4.27	11.19
Sinai Reli	6.61	1.76	3.16	10.07
Jamaica	5.66	1.13	3.44	7.87
Lunga Lunga Center	10.99	1.73	7.60	14.38
Milimani	18.05	2.31	13.52	22.58
Donholm	2.64	0.88	0.92	4.37
Riverside	4.29	1.07	2.19	6.38
Kingstone	3.91	0.89	2.15	5.66
Uchumi	10.52	2.18	6.24	14.80
*Villages in Korogocho*	(*p* < 0.001)			
Korogocho B	16.96	2.73	11.62	22.31
Korogocho A	9.28	1.63	6.08	12.48
Grogon A	9.97	2.34	5.38	14.55
Grogon B	9.76	2.80	4.26	15.25
Gitathuru	10.54	1.80	7.01	14.06
Highridge	13.81	1.55	10.76	16.85
Nyayo	4.20	2.10	0.09	8.31
Kisumu Ndogo	8.01	1.66	4.75	11.26

$\hat{\theta }$, prevalence; σ, standard error for the prevalence estimate; data in parenthesis are *p*-values; LCB, 95% lower class boundary; UCB, 95% upper class boundary.

**Figure 2:**
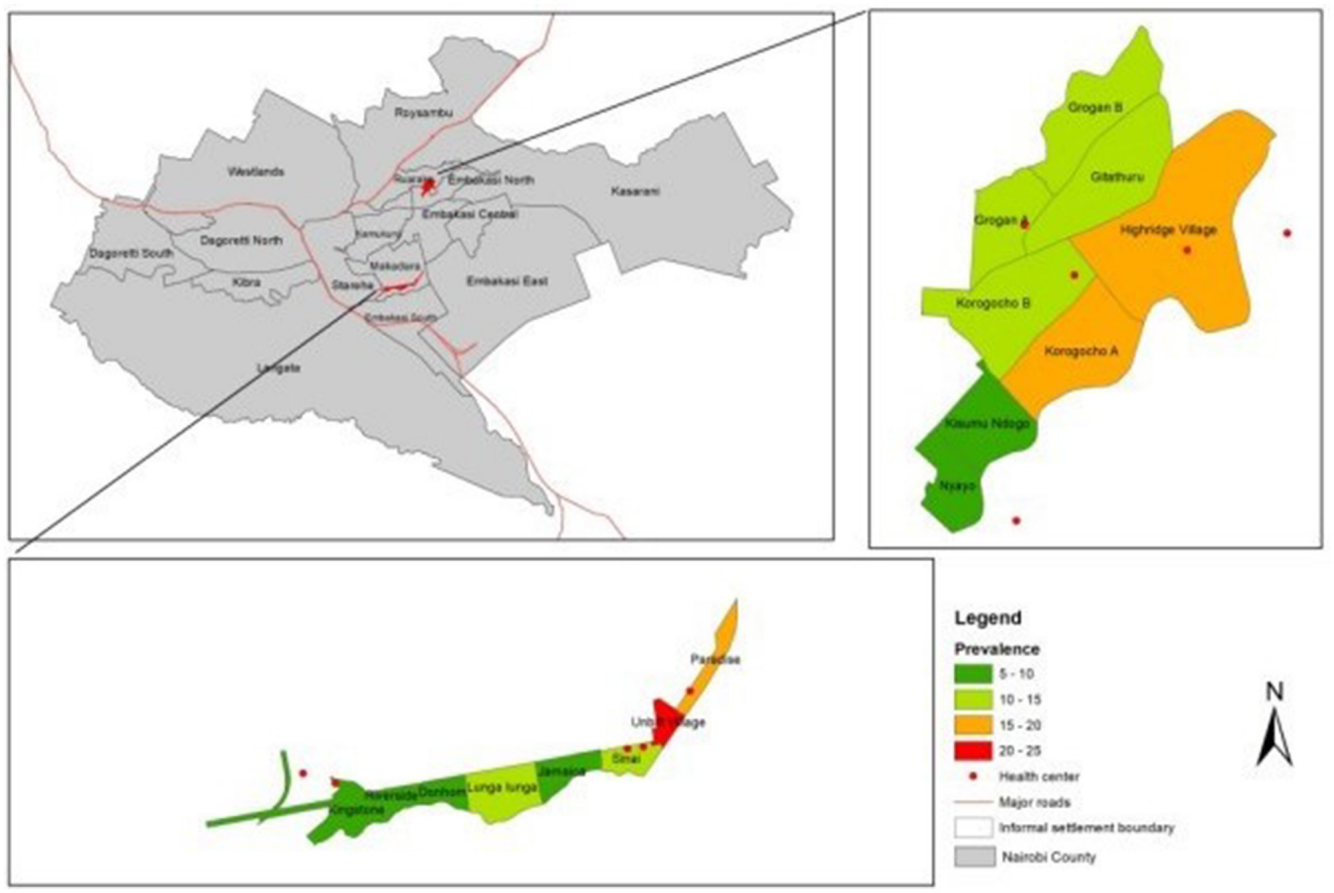
Clustering of epilepsy by site.

The overall crude prevalence of epilepsy was 9.40 per 1,000 people (95% CI 8.60–10.20). Prevalence was higher in Korogocho at 11.15 cases per 1,000 people (95% CI 9.75–12.56) than Viwandani at 8.32 cases per 1,000 people (95% CI 7.36–9.27), *p* < 0.001. There was great variability in prevalence by site (Figure 2), especially in Viwandani with a large range of 20.41 between the village with lowest prevalence (Donholm: prevalence = 2.64/1,000, 95% CI 0.92–4.37) and the highest prevalence (Paradise B: prevalence = 23.05/1,000, 95% CI 16.07–30.02), *p* < 0.001. Though relatively lower than Viwandani, there was also significant variation in Korogocho with a range of 12.76 from the village with the lowest prevalence (Nyayo: prevalence = 4.20/1,000, 95% CI 0.09–8.31) to highest prevalence (Korogocho B: prevalence=16.96/1,000, 95% CI 11.62–22.31), *p* < 0.001.

Table 3 presents prevalence estimates grouped by the field interviewer who screened the participant in the first stage. While interviewer variations may be outside the control of analysts and researchers, this analysis focuses on detecting how such variations or clustering may affect estimation of prevalence. For instance, if a field interviewer erroneously screens a patient to be negative when in fact should have been positive, then that participant will be erroneously excluded from the computation of prevalence, hence underestimating prevalence. Further, if a participant declines to participate in the study as a result of interviewer related effects, then this may contribute to non-response or attrition which may influence prevalence.

**Table 3:** Prevalence grouped by the interviewer who screened the participant in the first stage.

	**$\hat{\theta }$/1,000**	**σ**	**LCB**	**UCB**
*Interviewers in Viwandani*	(*p* < 0.001)			
Interviewer 1 (F)	8.92	2.46	4.09	13.74
Interviewer 3 (F)	9.72	2.28	5.25	14.20
Interviewer 8 (F)	1.11	0.79	–0.43	2.66
Interviewer 10 (F)	2.07	1.03	0.04	4.10
Interviewer 11 (F)	4.62	1.46	1.76	7.47
Interviewer 13 (F)	3.66	1.38	0.95	6.37
Interviewer 14 (F)	1.93	0.86	0.24	3.62
Interviewer 16 (F)	20.06	2.78	14.61	25.50
Interviewer 17 (F)	10.86	2.25	6.44	15.27
Interviewer 18 (F)	11.49	4.32	3.02	19.97
Interviewer 2 (M)	5.68	1.79	2.17	9.19
Interviewer 4 (M)	11.11	2.05	7.09	15.13
Interviewer 5 (M)	4.38	1.65	1.14	7.62
Interviewer 6 (M)	21.73	3.20	15.45	28.01
Interviewer 7 (M)	9.63	2.20	5.32	13.95
Interviewer 9 (M)	10.80	2.03	6.82	14.78
Interviewer 12 (M)	3.40	1.28	0.89	5.92
Interviewer 15 (M)	3.60	1.80	0.08	7.13
*Interviewers in Korogocho*	(*p* < 0.001)			
Interviewer 19 (F)	11.49	2.44	6.72	16.27
Interviewer 20 (F)	10.86	2.48	6.00	15.72
Interviewer 22 (F)	8.42	2.16	4.17	12.66
Interviewer 28 (F)	6.48	2.04	2.47	10.48
Interviewer 21 (M)	4.96	1.65	1.73	8.20
Interviewer 23 (M)	7.19	2.54	2.22	12.16
Interviewer 24 (M)	10.99	2.33	6.42	15.56
Interviewer 25 (M)	14.57	2.84	9.01	20.13
Interviewer 26 (M)	14.77	2.82	9.24	20.30
Interviewer 27 (M)	5.79	1.49	2.87	8.71
Interviewer 29 (M)	23.70	3.16	17.51	29.89
Interviewer 30 (M)	11.18	3.35	4.61	17.75

Average prevalence estimates by: Viwandani female interviewers: 7.44; Viwandani *male* interviewers: 8.79.

Korogocho female interviewers: 9.31; Korogocho male interviewers: 11.64.

F, female; M, male; $\hat{\theta }$, prevalence; σ, standard error for the prevalence estimate; data in parenthesis are *p*-values; LCB, 95% lower class boundary; UCB, 95% upper class boundary.

Similar to the site and village variations earlier described, results show that prevalence varied based on the interviewer (*p* < 0.001). Variation was higher among interviewers that worked in Viwandani than among those that worked in Korogocho, and also varied by the gender of the interviewer. For instance, the average of the prevalence estimates from female interviewers in Viwandani was lower at 7.44 compared to male interviewers in the same site at 8.79 (*p* < 0.001). Similar pattern is observed in Korogocho where prevalence from female interviewers was lower at 9.31 compared to male interviewers in the same site at 11.64 (*p* < 0.001). If we assume independence within the site and by gender of the interviewer, then there should be no significant difference between the average estimate by the gender of the interviewer. Any differences could be driven by clustering which can be both site specific, interviewer-related characteristics or both.

#### 3.1.2 Clustering and attrition effects on prevalence estimates under MAR mechanism

**Figure 3:**
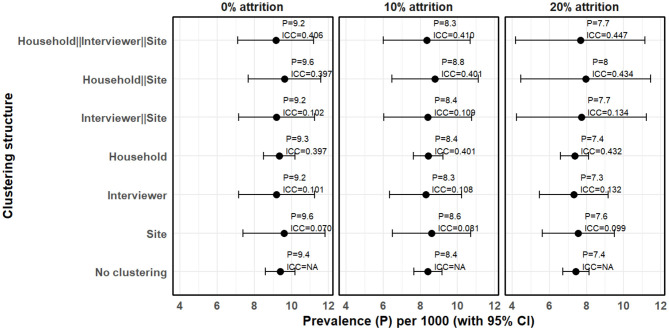
Clustering and attrition effects on prevalence estimates under MAR mechanism when attrition is not taken into account (complete case analysis).

Figure 3 presents intraclass correlation for data with no attrition (0% attrition) and with 10% and 20% attrition. The focus of this analysis is to examine how the prevalence estimate and its 95% confidence interval varies when different levels of clustering and attrition are considered but not addressed.

Assuming no attrition (0%) and that observations are independent (assuming no clustering), prevalence was estimated to be 9.40 cases per 1,000 people (95% CI 8.60–10.20). However, where random effects model was estimated, we observed substantial clustering at multiple levels. Household level clustering was high with ICC of 0.397 followed by interviewer level clustering with ICC of 0.101 and site level clustering with ICC of 0.070. Hierarchical model with only site and interviewer had an ICC of 0.102 but when household clustering was included to make it three-level hierarchical model, the ICC increased to 0.406. Consequently, prevalence of epilepsy adjusted for hierarchical clustering where households are nested within interviewers and interviewers within sites was 9.15 cases per 1,000 people (95% CI 7.11–11.20). We also observe that interviewer level clustering can not be ignored. For instance, assuming no attrition, when only household and site level clustering were considered, the ICC was 0.398 and the prevalence of epilepsy was 9.63 cases per 1,000 people (95% CI 7.68–11.58), which is slightly higher than when all the three levels are considered. Generally, the standard errors increased when clustering is taken into account resulting in wider confidence intervals. It was also observed that the confidence interval for household level clustering did not significantly vary from when household level clustering was not considered. While it is expected that a large ICC inflates the standard errors resulting to a wider confidence intervals, number of household clusters was large and thus the change in the standard errors was relatively minimal.

Attrition did not significantly affect intraclass correlation coefficient but the prevalence estimates and confidence intervals generally varied when different levels of attrition and clustering was considered. For instance, at 10% attrition, the multilevel model where households are nested within interviewers and interviewers with sites, yielded a prevalence estimate of 8.35 cases per 1,000 people (95% CI 6.01–10.69), a standard error of 1.19 with an intraclass correlation of 0.410. This implies a difference between upper and lower 95% confidence boundaries of 4.68, which is significantly higher than 1.51 when clustering is not taken into account. At 20% attrition, the difference between the two 95% confidence boundaries increased to 6.98 and the prevalence estimate reduced to 7.67 cases per 1,000 people (95% CI 4.18–11.16). This means that in a population-based survey, if not taken into account, attrition can lead to an underestimation of prevalence, and clustering can lead to underestimation of standard errors leading to deflated confidence intervals.

**Figure 4:**
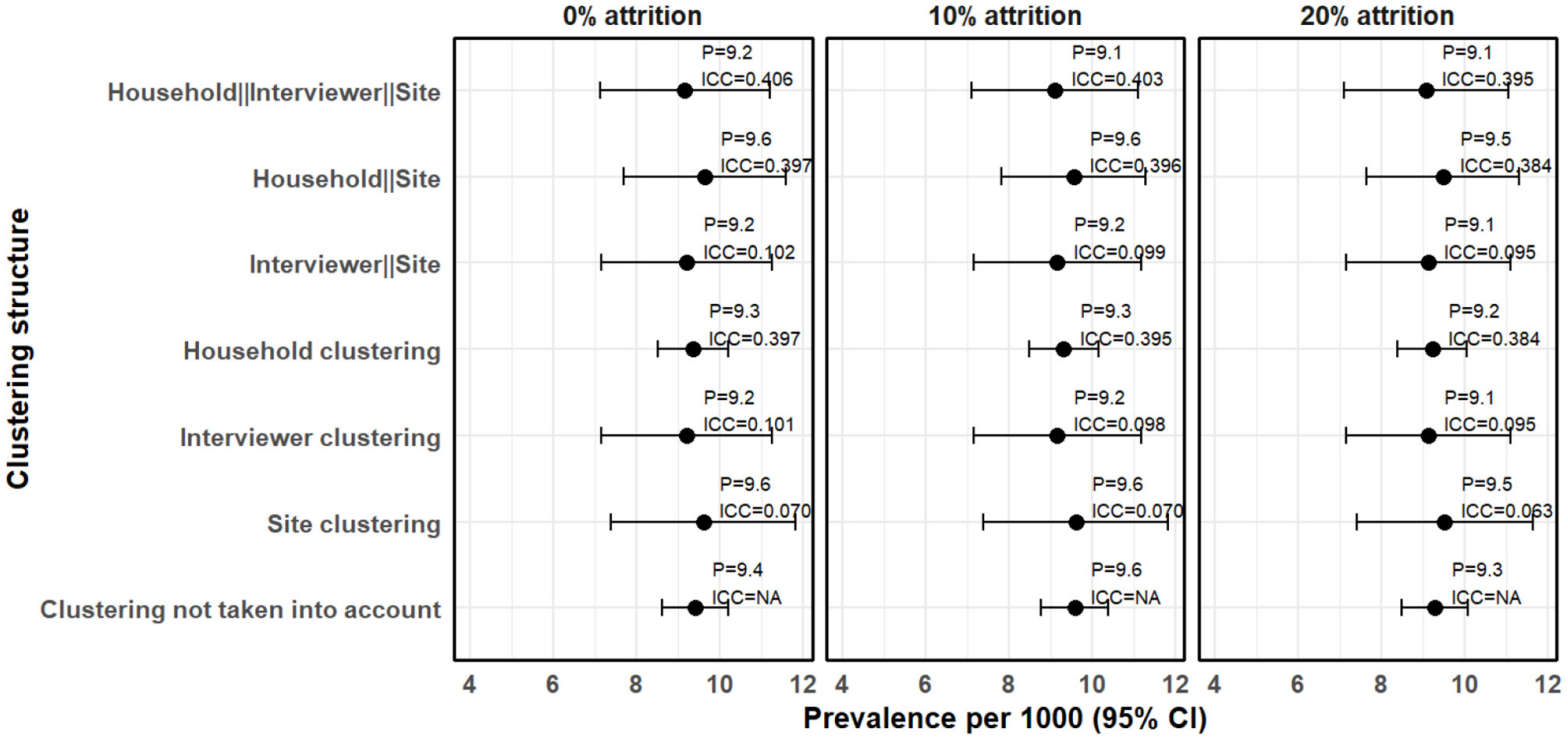
Clustering effects on prevalence estimates when the missing data due to attrition are imputed.

We also examined how the estimates and the standard errors changed when attrition is taken into account. Figure 4 shows the clustering effects on the prevalence estimates, standard errors and ICC when missing data due to attrition are imputed.

In Figure 4, the missing data due to attrition were imputed using the sKNN method. After accounting for 10% attrition, the estimated prevalence increased slightly to 9.58 cases per 1,000 people (95% CI 8.77–10.38) without clustering adjustment, compared to 8.40 cases per 1,000 people (95% CI 7.64–9.16) when the missing data is not imputed (complete case analysis). Clustering effects remained evident, with household-level clustering still the highest (ICC = 0.402), followed by interviewer-level clustering (ICC = 0.109) and site-level clustering (ICC = 0.081). The three-level hierarchical model, where households are nested within interviewers and interviewers nested within sites, resulted in a prevalence estimate of 9.10 cases per 1,000 people (95% CI 7.10–11.10). The resultant ICC was 0.403, which is not significantly different from the 0.410 observed when complete case analsysis was used. This means that imputation preserved the clustering structure of the dataset.

After accounting for the 20% attrition, prevalence estimates remained higher than those obtained using complete case analysis. Similarly, the ICC values remained stable after imputation. The three-level hierarchical model resulted in a prevalence estimate of 9.07 cases per 1,000 people (95% CI 7.11–11.04), with an ICC of 0.395, which is slightly lower than that obtained using complete case analysis (0.447). This means that larger values of missing if not taken into account may increase ICC. Furthermore, confidence intervals remained wider when clustering was taken into account, reinforcing the impact of hierarchical structure on standard error estimation.

**Figure 5:**
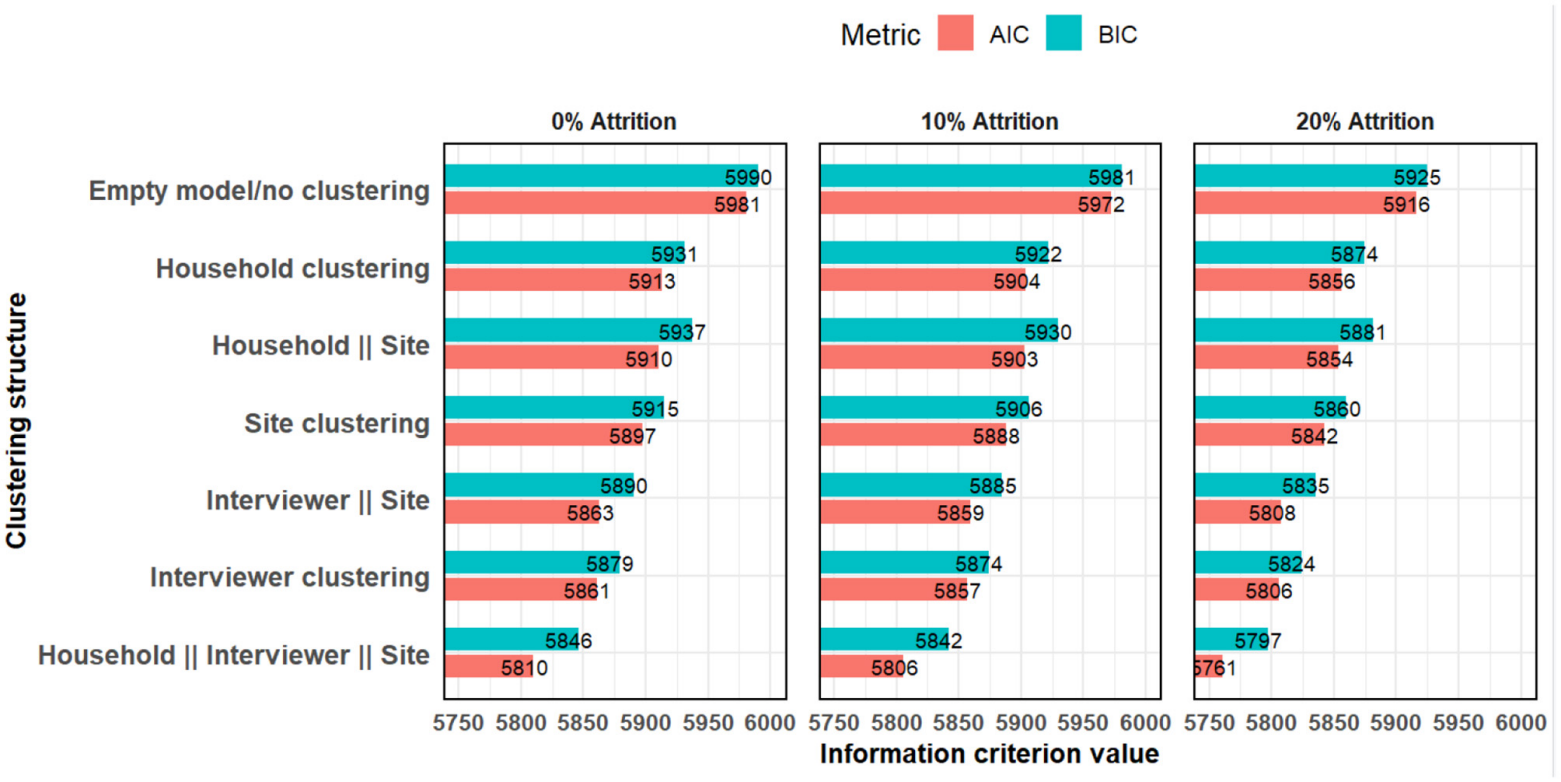
Akaike and Bayesian information criteria comparing models with various clustering structures.

For model diagnostics, the random effects models had lower AIC and BIC across all the various clustering structures (Figure 5). This indicates that the random effects model is a better fit.

### 3.2 Discussion

We examined how clustering affects estimation of standard errors for prevalence estimates. We found significant variations the prevalence estimates when grouped by the interviewers who screened the individuals in the first round of the survey. This could be due to variations in training, experience or individual interviewer characteristics, which may influence how they administer the questionnaires. In our study, the field interviewers were not specialists in epilepsy diagnosis but were trained about how to administer the questionnaire to screen for possible cases at the first stage. Confirmation of diagnosis was done by a trained neurologist. Any variability on how the field interviewers administered the questionnaire may introduce inconsistency response rates and in the final estimates. Our previous analysis of prevalence of epilepsy based on the same dataset, and other similar studies on epilepsy prevalence in similar settings (Ngugi et al., 2013; Kariuki et al., 2021) did not consider the various levels of clustering considered in this paper (Mwanga et al., 2024).

Substantial intraclass correlation coefficients (ICCs) at the household, interviewer, and site levels confirm the presence of hierarchical structure in the data. Ignoring such clustering can result in underestimated standard errors and biased prevalence estimates (Snijders and Bosker, 2011). This aligns with prior studies emphasizing the need to account for interviewer effects and geographic clustering to ensure valid estimates (Olson et al., 2020; Lipps and Lutz, 2017; Harling et al., 2019).

The high household-level clustering suggests that individuals within the same household had similar responses. This is likely due to shared environmental, genetic (Wang et al., 2017; Ottman et al., 1996), or socio-economic factors that influence epilepsy prevalence (Li et al., 2008; Ngongo et al., 2015). Similarly, the presence of interviewer effects reinforces the need for standardized interviewer training, supervision and careful monitoring during data collection. Differences in interviewer demeanor, skill level, and interpretation of survey questions can substantially influence self-reported data. Prior studies (Lipps and Lutz, 2017) and recent work in Kenya (Gachau, 2021) have stressed the need to account for hierarchical effects–especially interviewer or physician-level clustering–when analyzing survey and clinical data. Implementing rigorous interviewer training protocols and pilot testing can mitigate these biases.

Consistency of responses by interviewers has been emphasized by our study. The importance of rigorous interviewer training and pilot testing to reduce bias is well established. Large-scale surveys such as the Demographic and Health Surveys (DHS) employ detailed interviewer manuals, detailed training of the interviwers that last several days, and supervised field-based pilot interviews before commissioning the interviewers to the field to collect data (ICF International, 2012). Interviewers are often certified through competency assessments before deployment. These practices provide a useful model for future prevalence studies involving self-reported health outcomes, such as epilepsy diagnosis, where interviewer consistency is critical for data quality.

In this study, clustering generally increased the width of confidence intervals for the parameter estimates. Clustering effects also varied by the type of cluster, with clustering at village and household levels showing higher prevalence estimates and wider confidence intervals. Hierarchical models resulted in moderate increased standard error and hence wider confidence interval. The findings are similar to those previously documented in literature, such as those by Turner et al. (2001) and Thompson and Syed (2012), who demonstrated that failure to account for clustering underestimates standard errors, inflates type I error rates, and leads to misleading conclusions. This emphasizes the need for appropriate statistical methods that account for clustering effects, especially in hierarchical clustered survey data. Studies such as Murray et al. (2004) and Campbell et al. (2004) have also confirmed the substantial impact of clustering in randomized trials and prevalence surveys, underscoring the importance of multilevel modeling in health research.

In addition to clustering, attrition also poses a challenge in estimating prevalence. In our analysis, attrition was addressed using ML-based imputation methodology. While imputing missing data helps to account for attrition, the ideal practice is to minimize attrition by desiging a longitudinal or multi-stage study with measures to improve response rates in subsequent timepoints. In our study, the persistence of relatively high ICC values after imputation suggests that the hierarchical structure of the data was preserved. Importantly, the inclusion of random effects and ICCs in our model not only accounts for correlation within clusters but also provids more accurate standard errors and confidence intervals for prevalence estimates. Previous research by Little and Rubin (2002) has shown that unaddressed missing data can lead to bias and reduced efficiency in prevalence estimates. Their work supports our approach of ensuring the missing data are imputated using appropriate methods to adjust for potential biases due to nonresponse. Taken together, these findings reinforce the importance of accounting for both clustering and attrition when designing and analyzing survey data to enhance the robustness, validity and reliability of the estimates.

This study has strengths. First, it is a population-based survey from a well characterized setting from the Nairobi Urban Health and Demographic Surveillance System, which enhanced the generalizability of the findings to the broader population in informal settlements of Nairobi. Second, it incorporates advanced statistical methods, including mixed-effects models and machine learning-based imputation techniques, to address clustering and attrition, which are common in survey data. Third, the study provides a practical demonstration of how site, interviewer and household-level clustering can be taken into account in the analysis of self-reported outcomes and providing suggestings for future research on similar topics.

A few limitations exist. First, the study does not have a comparative arm to objectively evaluate the various approaches for enhancing accuracy of estimates. The study relies on statistical modeling techniques to adjust estimates. Second, the analysis for missing data assumes a missing at random (MAR) mechanism, which may not fully capture the complexity of real-world missing data patterns. Third, while interviewer effects were modeled statistically, the study did not conduct a using a randomized trial to directly assess potential interviewer characteristics such as experience, gender, age or training level, that could have influenced the observed clustering. Future studies on how to enhance consistency among interviewers should consider collecting these information from the interviewers to enable proper analysis of any potential biases related to interviewer effects. Fourth, despite efforts to minimize these through standardized questionnaires, interviewer training and statistical modeling as demonstrated in this study, epilepsy diagnosis also relies on other factors such as participant recall bias which has not been considered in this study.

In conclusion, addressing clustering and attrition effects is essential for producing accurate and reliable prevalence estimates in population-based surveys. Survey designs should incorporate strategies to minimize biases related to these issues. Consistency among interviewers for self-reported outcomes can be enhanced by rigorous training and pilot testing before the actual surveys are conducted. Employing robust statistical methods and transparent reporting enhances the validity of self-reported data and inform evidence-based public health interventions. We recommend that during design and preparation for data collection, randomize interviewers across sites and collect metadata on interviewer characteristics to facilitate adjustment. Further, attrition bias can be minimized by conducting targeted mobilization and follow-ups to improve response rates which are proven approaches based on previous studies (Teague et al., 2018), and accounting for it using standard statistical approaches such multiple imputation or machine learning-based imputation methods.

## Data Availability

The data analyzed in this study is subject to the following licenses/restrictions: Creative commons attribution CC-BY-NC-4.0. Requests to access these datasets should be directed to corresponding author (dmwanga@aphrc.org), the principal investigators (gasiki@aphrc.org or charles.newton@psych.ox.ac.uk) or access through https://microdataportal.aphrc.org/index.php/catalog after request approval.

## Group members of EPInA Study Group

Abankwah Junior, Albert Akpalu, Arjune Sen, Bruno Mmbando, Charles R. Newton, Cynthia Sottie, Dan Bhwana, Daniel Mtai Mwanga, Damazo T. Kadengye, Daniel Nana Yaw, David McDaid, Dorcas Muli, Emmanuel Darkwa, Frederick Murunga Wekesah, Gershim Asiki, Gergana Manolova, Guillaume Pages, Helen Cross, Henrika Kimambo, Isolide S. Massawe, Josemir W Sander, Mary Bitta, Mercy Atieno, Neerja Chowdhary, Patrick Adjei, Peter O. Otieno, Ryan Wagner, Richard Walker, Sabina Asiamah, Samuel Iddi, Simone Grassi, Sloan Mahone, Sonia Vallentin, Stella Waruingi, Symon Kariuki, Tarun Dua, Thomas Kwasa, Timothy Denison, Tony Godi, Vivian Mushi, William Matuja.
